# Relating Structural and Optical Properties of Organic Thin Films on Chemically Inert Substrates

**DOI:** 10.1021/acsomega.5c03920

**Published:** 2025-09-26

**Authors:** Nina Kainbacher, Peter Puschnig, Oliver T. Hofmann

**Affiliations:** † Institute of Physics, 27267 University of Graz, NAWI Graz, Universitätsplatz 5, 8010 Graz, Austria; ‡ Institute of Solid State Physics, 27253 Graz University of Technology, NAWI Graz, Petersgasse 16/II, 8010 Graz, Austria

## Abstract

Molecular monolayers on a supporting substrate can order in different configurations, i.e., polymorphs. These different polymorphs can also exhibit distinctly different optical properties owing to variations of the intermolecular coupling between the transition dipole moments, which in turn may affect transition energies and oscillator strengths. In this study, we computationally investigate the impact of polymorphism of organic molecular monolayers on optical absorption spectra using a combination of machine-learning-assisted structure search and density functional theory. Specifically, we systematically detail various influencing factors, including the geometric distortions upon adsorption, interactions between transition dipole moments, and changes in selection rules due to variations in symmetry of different polymorphs. As an example, we predict the polymorphism of the dipolar organic molecule 2-nitro-pyrene-7-amine on NaCl(100) using a machine learning-based structure search and calculate its optical properties based on density functional theory and the random phase approximation. Specifically, we find two different polymorphs where the lowest excitation energy significantly differs by approximately 0.2 eV.

## Graphical abstract

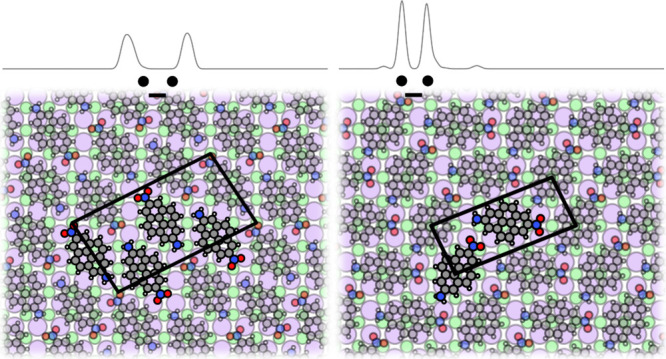

## Introduction

Molecular thin films and monolayers on a supporting substrate can order in different configurations, i.e., polymorphs, which exhibit distinctly different optical properties (e.g., absorption energies, oscillator strengths, etc.).^1–4^ These two-dimensional layers show fascinating properties, as, for example, superradiance^5–7^ and switching between H- and J-aggregates by different brick layer geometries.^8^ Being able to understand the connection between these structural and optical characteristics of a polymorph is a particularly important aspect for the field of thin-film organic optoelectronics, as it allows designing molecular monolayers with specific optical properties,^9–11^ such as optical gaps that are close to the ideal value for the Schottky–Queisser limit.

In a simplified picture, i.e., neglecting depolarization effects, the interplay between polymorphism and optical properties^12–14^ is usually attributed either to intermolecular rehybridization (as, e.g., in the case of quinacridone^15^ or 5-methyl-2-[(2-nitrophenyl)­amino]-3-thiophenecarbonitrile (ROY)^16–19^ ), or to the coupling of the molecular states within Kasha theory for molecular dimers, also known as H- and J-aggregate theory.^20,21^ This theory has been extended to one-dimensional structures (i.e., molecular chains),^22,23^ as well as two-dimensional molecular monolayers on insulators.^8,22,24–27^ To affect the optical properties of thin films, so far the focus has largely been laid on chemical modifications of the molecules, e.g., by endowing them with different molecular chains^23^ or by choosing derivatives of one specific molecular core.^28^ However, the change of the molecular coupling is not the only aspect and, as we will show in this work, sometimes not even the largest factor that affects polymorphism-induced optical changes.

Especially for thin films, the fact that the molecules can adopt different adsorption sites on the substrate can induce substantial changes in the optical spectra. Moreover, depending on the strength and the nature of the intermolecular interaction, different polymorphs may display different degrees of localization of the molecular orbitals, i.e., in one polymorph, the orbitals may remain confined to individual molecules, while in another, the same state may become delocalized over the whole layer, depending on the symmetry of the unit cell and the strength of the intermolecular coupling.

In this work, we employ density functional theory to exemplify the extent of these effects for two prototypical polymorphs of a monolayer 2-nitro-pyrene-7-amine (CAS registry number 126948-22-9, structure shown in Figure 1) adsorbed on NaCl(100). We focus on a strongly dipolar molecule, since dipole–dipole interactions constitute both a major driving force for the formation of different molecular arrangements^29–33^ and are frequently invoked to explain changes in the optical spectra.^20,21^ Moreover, donor–acceptor-substituted molecules like 2-nitro-pyrene-7-amine are of profound technological relevance in optoelectronic applications, such as dye-sensitized solar cells,^34^ organic light-emitting devices,^35^ or in the field of nonlinear optics.^36^ The choice of NaCl as a substrate is motivated by its frequent use as a substrate in surface science, especially when electronic decoupling from underlying metallic substrates is desired.^37–40^

**Figure 1:**
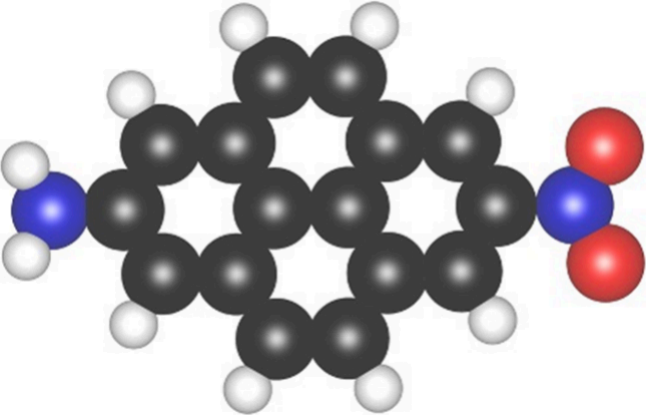
Structure of the dipolar molecule 2-nitro-pyrene-7-amine. Carbon atoms are colored in black, hydrogen in white, oxygen in red, and nitrogen in blue.

## Results and Discussion

To predict polymorphs for the monolayer of nitropyreneamine on NaCl(100), we employ the machine learning-based structure search algorithm SAMPLE,^41^ which is explained in the theoretical methods. Through this structure search, we find two different prototypical polymorphs with the same coverage (i.e., number of molecules per area): A brickwall (***BW***) phase with antiparallel stripes consisting of flat-lying molecules arranged in a head-to-tail configuration (see Figure 2a) and a herringbone (***HB***) phase with flat-lying molecules arranged almost perpendicular to each other’s neighbors (see Figure 2b). We characterize their optical properties by computing the imaginary component of the dielectric function within the random phase approximation (RPA). While this is common practice when aiming at a comparison with experimental absorption spectra,^42^ we note that the actual optical absorption coefficient depends on both the imaginary and real parts of the dielectric function. A direct comparison with measured optical density would be furthermore obfuscated by other experimental effects such as reflection and interference. For the sake of clarity, therefore, we refer to our calculated spectra as the “imaginary part of the dielectric function” instead and the peaks as “optically active transitions.”

**Figure 2:**
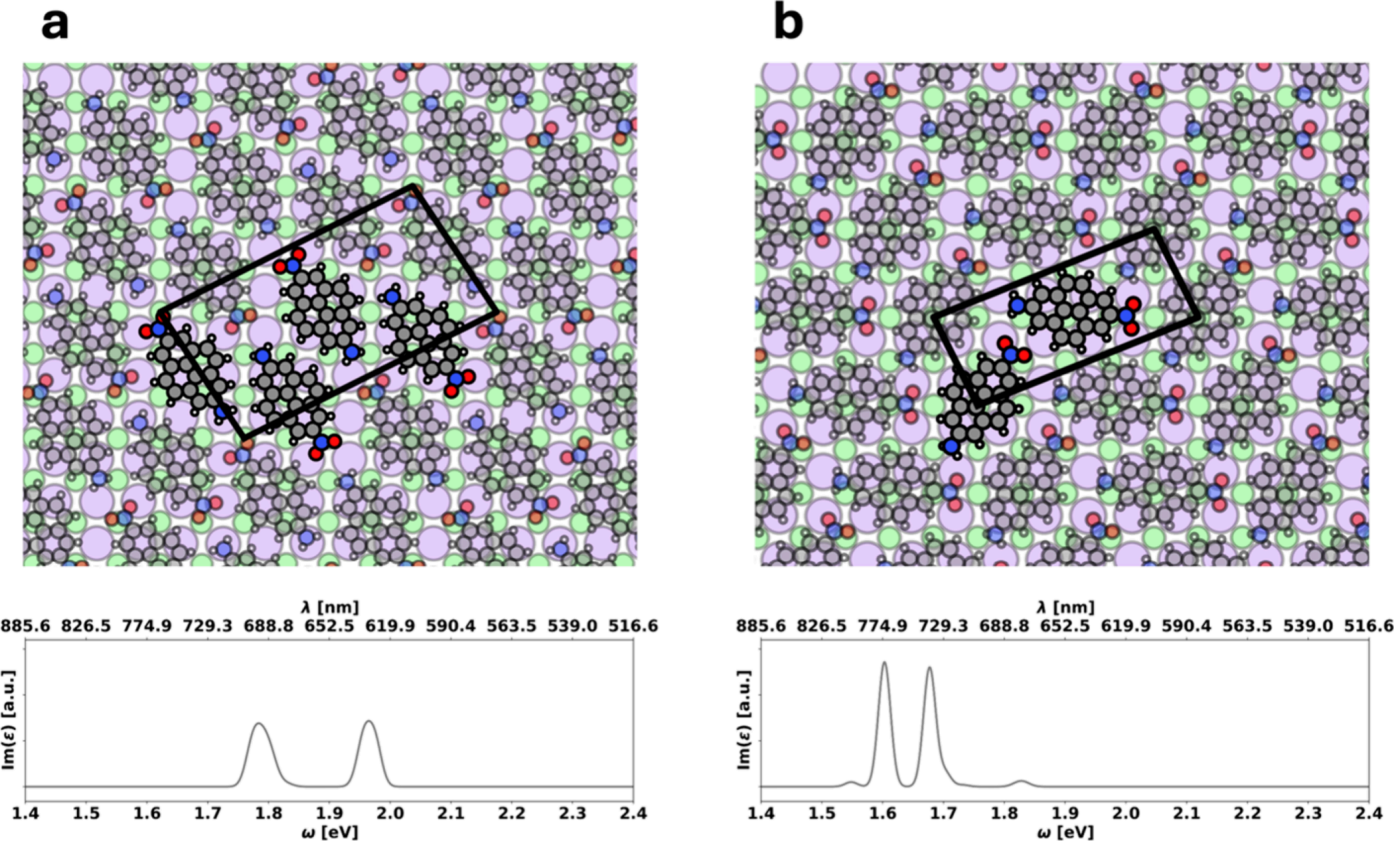
Two polymorphs of monolayers of nitropyreneamine on NaCl(100). The unit cell is marked in black, and the contained molecules are highlighted. Panel a shows the brickwall (***BW***) and panel b the herringbone (***HB***) phase, together with their unpolarized absorption spectrum in the lower panel.

We find the first notable peak of the imaginary part of the dielectric function at around 1.8 eV (689 nm) for the ***BW*** (Figure 2a, lower panel) and at around 1.6 eV (775 nm) for the ***HB*** phase (Figure 2b, lower panel), i.e., the polymorphism causes a quite substantial shift of 0.2 eV. Both values are substantially red-shifted relative to the first optically active transition in the gas phase, which we find at around 2.3 eV (654 nm) (see Figure S1c, Supporting Information).

To understand where the large shift comes from, it is instructive to first highlight two salient structural differences between the phases: (i) the adsorption sites of the molecules and (ii) the symmetry of the unit cell. The ***BW*** phase includes four molecules in the unit cell, with two molecules each being symmetry-equivalent by inversion symmetry. We enumerate the two nonequivalent adsorption sites with the Roman numerals I and II, respectively (see Figure 3a,b, and the distribution within the unit cell in Figure 3e). Conversely, the HB phase contains only two molecules per unit cell, with one adsorption site equivalent to II (also contained in the ***BW*** phase) and an additional adsorption site designated with the Roman numeral III. The different adsorption sites in the unit cell are depicted in Figure 3d. This phase shows neither inversion nor mirror or rotation symmetry.

**Figure 3:**
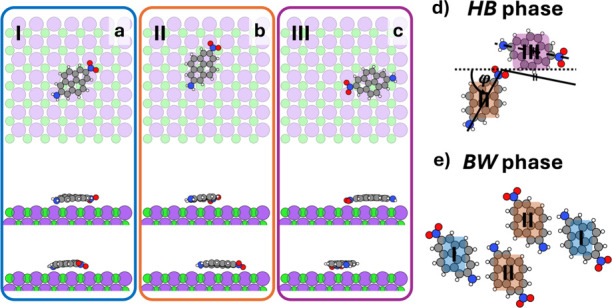
Visualization of (panel a) the adsorption geometries I (color-coded in blue), (panel b) II (orange), and (panel c) III (purple) on NaCl(100) (Na colored in purple and Cl in green) in the top view and two different side views. The two polymorphs, ***HB*** in (panel d) and ***BW*** phase in (panel e) shown on the right side are constructed from these adsorption geometries.

The three adsorption sites differ notably in the way the functional groups align relative to those of the NaCl surface atoms. Adsorption site I is characterized by the center of the molecule being between a Na and Cl atom, with the NH_2_-group almost on top of a Na atom and the NO_2_-group located such that one oxygen is near a Na and the other near a Cl atom (see Figure 3a). Adsorption site II is characterized by the center of the molecule being close to a Cl atom, with the NH_2_-group in between Na and Cl atoms and the NO_2_-group, again, located such that one oxygen atom is close to a Na and the other close to a Cl atom (Figure 3b). As depicted in Figure 3c, adsorption site III is characterized by the center of the molecule close to a Cl atom, the NH_2_-group close to a Na atom, and the NO_2_ group located such that both oxygen atoms are in close proximity to Na atoms. We note that due to the size of the molecule relative to the NaCl lattice constant, it is impossible for the molecule to find “chemically intuitive” adsorption sites, where the negatively charged NO_2_ group sites on top of positively charged Na atoms and the positively charged NH_2_-group on top of negatively charged Cl atoms. Rather, all adsorption sites that individual molecules can adopt (see Supporting Information Figure S3 for a comprehensive visualization of stable adsorption sites and their relative energies) experience a trade-off between (un)­favorable molecule–substrate interactions. As we will see below, this has a major impact on the dielectric function.

Based on the concept of Davydov splitting, we could expect that the single optical transition of the gas phase at around 2.3 eV splits into multiple components due to (anisotropic) coupling of the transition dipole moments (transition dipoles are defined as ⟨ψ_f_| *er̂* | ψ_i_⟩, with ψ_f_ being one specific unoccupied orbital and ψ_i_ being one specific occupied orbital). At first glance, this indeed seems to be the case. As shown in the computed dielectric functions (depicted in the bottom panel of Figure 2), we observe a double-peak structure reminiscent of a splitting into an upper and a lower Davydov component. This splitting is about twice as large for the ***BW*** phase (ca. 0.2 eV) as for the ***HB*** phase (ca. 0.1 eV).

To test whether this is indeed Davydov splitting, it is instructive to analyze a spectrum where the polarization of the incoming light is along the different molecules’ long axes (see Figure 4). This is done by projecting I(ε) onto these directions. We note in passing that in the gas phase, the first optically active transition shows the largest absorption in this polarization direction (see Figure S1 in the Supporting Information). Following the Kasha model, if the splitting was indeed due to coupling of the transition dipole moments, then we would expect that the ***BW*** phase (which is structurally similar to J-aggregates) shows a vanishing intensity for one component and a strong intensity enhancement of the other component. Conversely, for the ***HB*** phase, where the two molecules are almost orthogonal relative to each other, this projection should yield approximately equal intensities for both bands. As Figure 4 shows, both phases defy this expectation. Rather, the ***BW*** phase shows equal intensities for both peaks, whereas the ***HB*** phase shows large intensity for one peak and almost vanishing intensity for the other peak, depending on the light polarization. This observation is clearly incompatible with Kasha-type physics, showing clearly that for a flat-lying monolayer of organic molecules, other effects must play a major role.

**Figure 4:**
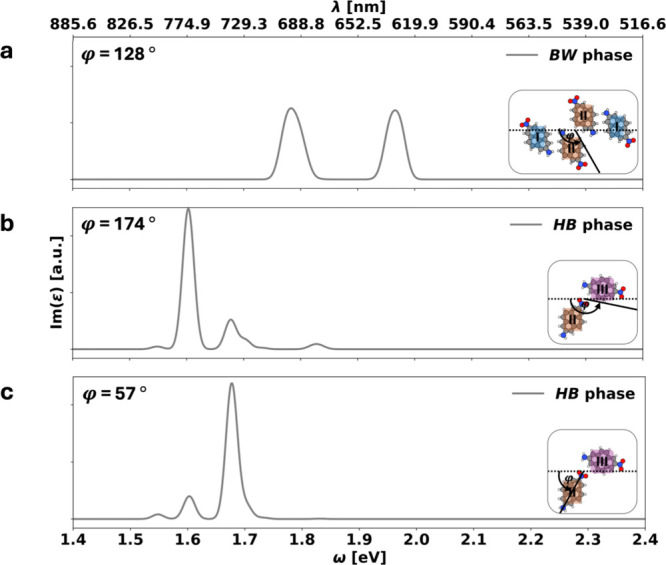
Imaginary part of the dielectric function ε plotted for the ***BW*** (panel a) and ***HB*** phases (panels b and c) on NaCl. For the ***HB*** phase, we show the phase for two polarizations due to the large difference in orientation of the two adsorption geometries III (purple) and II (orange) with φ = 57° and φ = 174°, respectively. The polarization angle φ is defined as shown in the illustrations on the bottom right of each plot and in Figure 3 in the illustration on the upper right.

The simplest explanation consistent with the findings above would be that the spectrum is, in fact, a combination of single-molecule contributions. More precisely, a plausible hypothesis would be that there is no significant intermolecular coupling, but rather, the different adsorption sites of nitropyreneamine result in structural and electronic changes that shift the optical transition from ∼2.3 eV in the gas phase to 1.6/1.7 eV for the ***HB*** phase and 1.8/2.0 eV for the ***BW*** phase. To test this hypothesis, we computed the optical transitions for isolated nitropyreneamine molecules on different adsorption sites I, II, and III.

As Figure 5b shows, the precise location on the surface has a tremendously large effect. Compared to the molecules in the gas phase (see Figure S1c in the Supporting Information), the largest shift is observed for III, where the first optically active transition is found at around 1.9 eV. Adsorption site II also experiences a large, but slightly smaller shift, and is found at around 2.0 eV. In salient contrast, for adsorption site I, the optical transition remains very close to the value of the free molecule in the gas phase and is found at around 2.2 eV.

**Figure 5:**
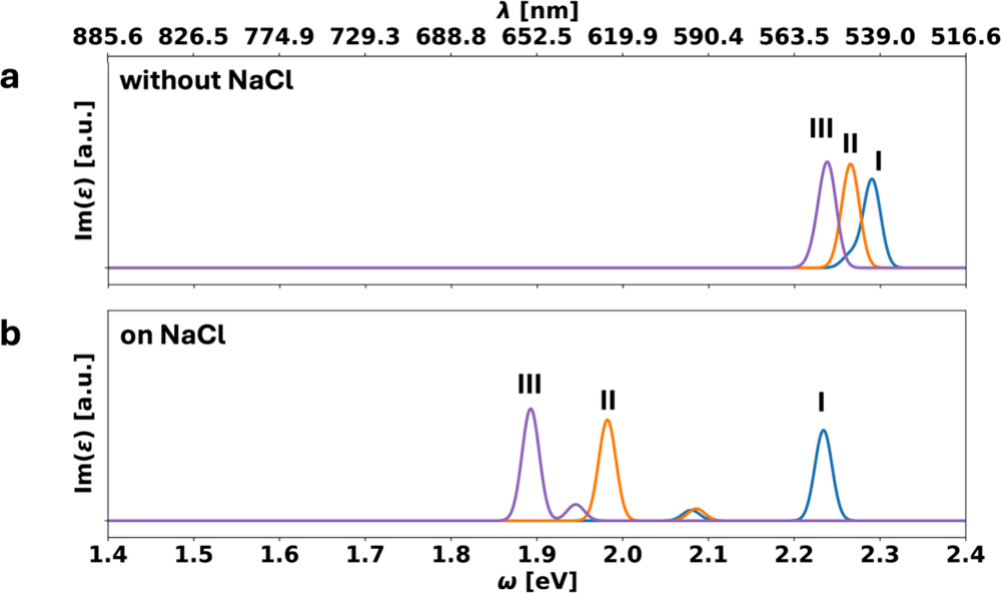
Imaginary part of the dielectric function Im­(ε) plotted for the adsorption sites I (blue), II (orange), and III (purple) in the gas phase (panel a) and on the NaCl(001) substrate (panel b). Note that the ***BW*** phase consists of adsorption sites I and II, and the ***HB*** phase consists of adsorption sites II and III.

There are, in principle, two different aspects that could give rise to these different shifts: On the one hand, the adsorption of the molecule on the surface is accompanied by a notable geometric relaxation, which most ostensibly (see Figure 3) lifts the planarity of the molecules and induces changes in the intramolecular bond lengths. On the other hand, the substrate electronically interacts with the molecule, perturbing the molecular orbitals and screening optical transitions. To differentiate between the two effects, we calculated the imaginary part of the dielectric function using the geometry the molecules adopt on the surface, but without the substrate present. We find (see Figure 5a) that geometric effects alone only account for minor shifts of a few 10 meV, i.e., despite the fact that NaCl is a very inert substrate, the geometric distortion has a markedly minor effect compared to the electronically induced shift.

To rationalize the electronic effect, it is instructive to inspect how the adsorption affects the orbital energies of nitropyreneamine. In the gas phase, the HOMO–1, which is localized on the aromatic core only, and the HOMO, which has a strong contribution to the NH_2_ group, are energetically degenerate. Upon adsorption, this degeneracy is lifted. As Figure 6 shows, compared to the gas phase, the HOMO is being stabilized the most for adsorption site I, presumably due to the favorable location of the NH_2_ group relative to a positively charged Na atom (cf. Figure 3). For II and III, where the NH_2_-group is located between Na and Cl (cf. Figure 3), the stabilization is much less pronounced. Conversely, the LUMO in the gas phase is nondegenerate and has a strong contribution to the NO_2_-group. For the adsorption site III, the favorable location of the NO_2_-group relative to the Na atoms leads to a strong stabilization of this orbital, which is absent for both I and II. As a result, on the surface, adsorption site I (experiencing the strongest stabilization for the occupied and the least stabilization for the unoccupied orbitals) yields the largest gap, whereas III (experiencing the strongest stabilization of the LUMO and the least stabilization of the occupied states) shows the smallest gap. We note that in all cases, the adsorption of the molecules leads to only minor changes of the orbital shapes compared to the gas phase, which can be explained by the above-described electrostatic (de)­stabilization of the electrons.

**Figure 6:**
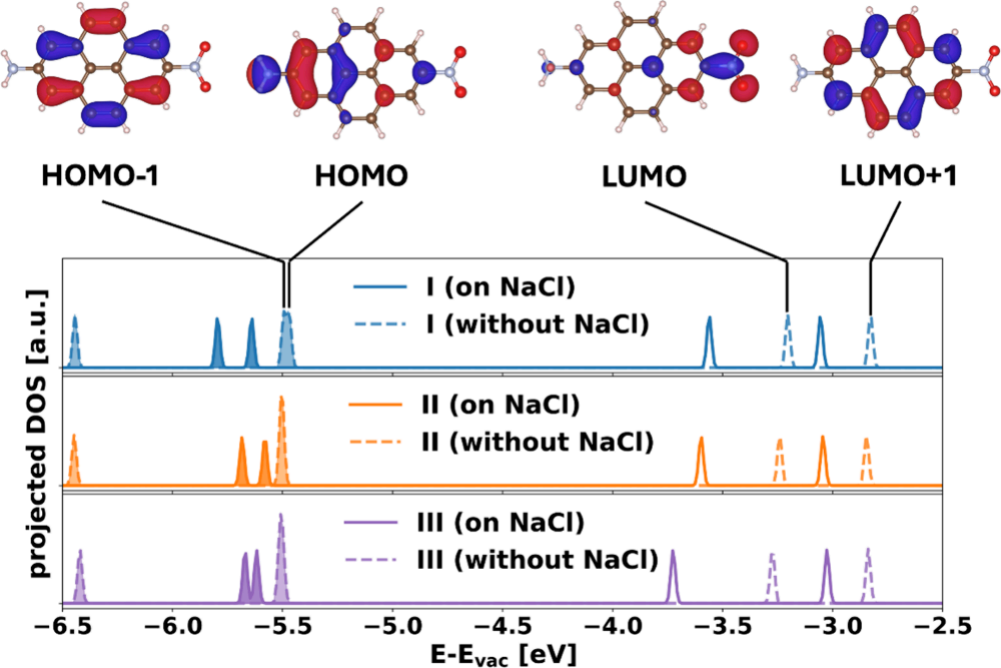
Upper panel: Molecular orbitals (HOMO–1, HOMO, LUMO, and LUMO+1) of the relaxed molecule in the gas phase. Lower Panel: Density of states (DOS) of the system projected onto the individual adsorption geometries on NaCl(100) (solid lines) and in the gas phase (dashed lines). The occupied states are indicated by the filled areas. Intensities of the peaks can only be compared within one DOS. Note that the valence band maximum of NaCl is found at ca. −6.6 eV and the conduction band at ca. −1.8 eV; i.e., both are outside of the plotted range of this figure.

The electronic interaction between NaCl and nitropyreneamine thus does explain the observed apparent splitting of the peaks in Figure 4: Adsorption site II, which has an intermediate gap, is present in both the ***BW*** and ***HB*** phases. Site I has a larger gap and is only present in the ***BW*** phase, while site III has the smallest gap and is only present in the ***HB*** phase. A posteriori, this finding is also consistent with the observation that we do not see a Kasha-type coupling of the transition dipole moment since Kasha theory implicitly assumes that the optical transitions of the coupled molecules are at (almost) the same energy. However, the electronic interaction between substrate and adsorbate does not suffice to explain the strong shift of the first optically active transition relative to the gas-phase molecule, which is significantly larger than 0.2 eV red-shifted in the closed layer compared to the isolated molecules on the surface. Clearly, this shift must originate from molecule–molecule interactions.

To understand the impact of these interactions, it is necessary to identify the participating molecular orbitals in each transition. This can be done by considering two aspects: (i) The density of states (DOS) of the combined systems projected onto the orbitals of the molecular film and (ii) the transition dipole moment between these orbitals which is calculated (in atomic units) as ⟨ψ_f_ | *r̂* | ψ_i_⟩, with ψ_f_ being one specific unoccupied orbital and ψ_i_ being one specific occupied orbital. The sum of squares of the transition dipole moments (more than one molecule can participate in a given transition) is directly proportional to the intensity of a given transition.

For the ***BW*** phase, we find that the first optically allowed transition (at ca. 1.8 eV) consists of a transition from the highest occupied state to the lowest unoccupied state of the molecular layer. Interestingly, as shown in Figure 7b, both states consist exclusively of orbitals localized on adsorption site II; hence, we denote it as HOMO­(II)→LUMO­(II) (i.e., molecular orbitals localized on the specific adsorption site). It is indicated by an orange arrow in the projected DOS in Figure 7a. The corresponding energy difference coincides with the transition energy of ca. 1.8 eV, as shown in Figure 4a. The participating molecular orbitals of the second optically allowed transition (shown in blue) are localized on both adsorption sites. In particular, HOMO–6­(I) and HOMO–7­(II) are mainly localized on I and II, respectively, and LUMO+1­(I) is localized on I, as shown in Figure 7c. It is indicated by the blue arrow in Figure 7a and can be identified in the imaginary part of the dielectric function (Figure 4a) as the second peak at around 2.0 eV. This explains the reason why there are two peaks of equal intensity when polarizing the absorption in the direction of the molecule’s long axis.

**Figure 7:**
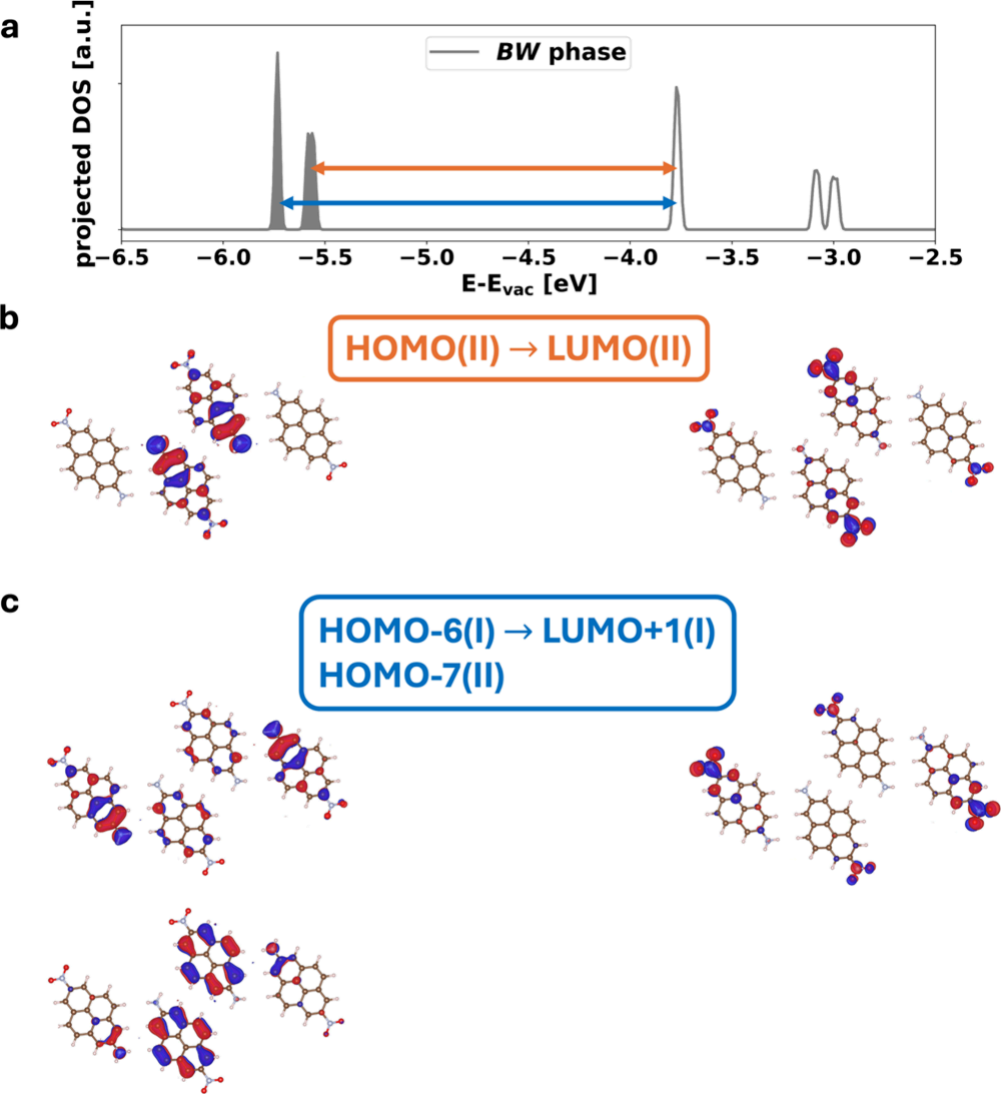
Panel a shows the density of states (DOS) of the system projected onto the molecular film of the ***BW*** phase on NaCl. The colored arrows correspond to the first two optically active transitions. The occupied states are indicated by the filled areas. Intensities of the peaks can be compared only within one DOS. Orange indicates the first transition from the HOMO­(II) to the LUMO­(II) localized on adsorption geometry II (panel b). The orbitals participating in the second transition are a transition from both HOMO–6­(I) and HOMO–7­(II), mainly localized on I and II, respectively, to the LUMO+1­(I), mainly localized on I (panel c) and indicated with a blue arrow in the projected DOS.

For the **HB** phase, the first optically allowed transition is a transition from HOMO–1­(III) to LUMO­(III) localized on adsorption geometry III, as shown in Figure 8b. It is indicated by a purple arrow in the projected DOS (Figure 8a). Therefore, the peak in Im­(ε) is more pronounced when the absorption is polarized in the direction of adsorption geometry III (see Figure 4b, polarization angle φ = 174°). The second optically active transition (as shown in Figure 4c, polarization angle φ = 57 °) is a transition from HOMO­(II) to LUMO+1­(II) localized on adsorption geometry II, as shown in Figure 8c and indicated by the orange arrow in the projected DOS (Figure 8a). The localization of the orbitals on the specific adsorption sites explains why the optical spectra cannot be understood solely by Kasha theory.

**Figure 8:**
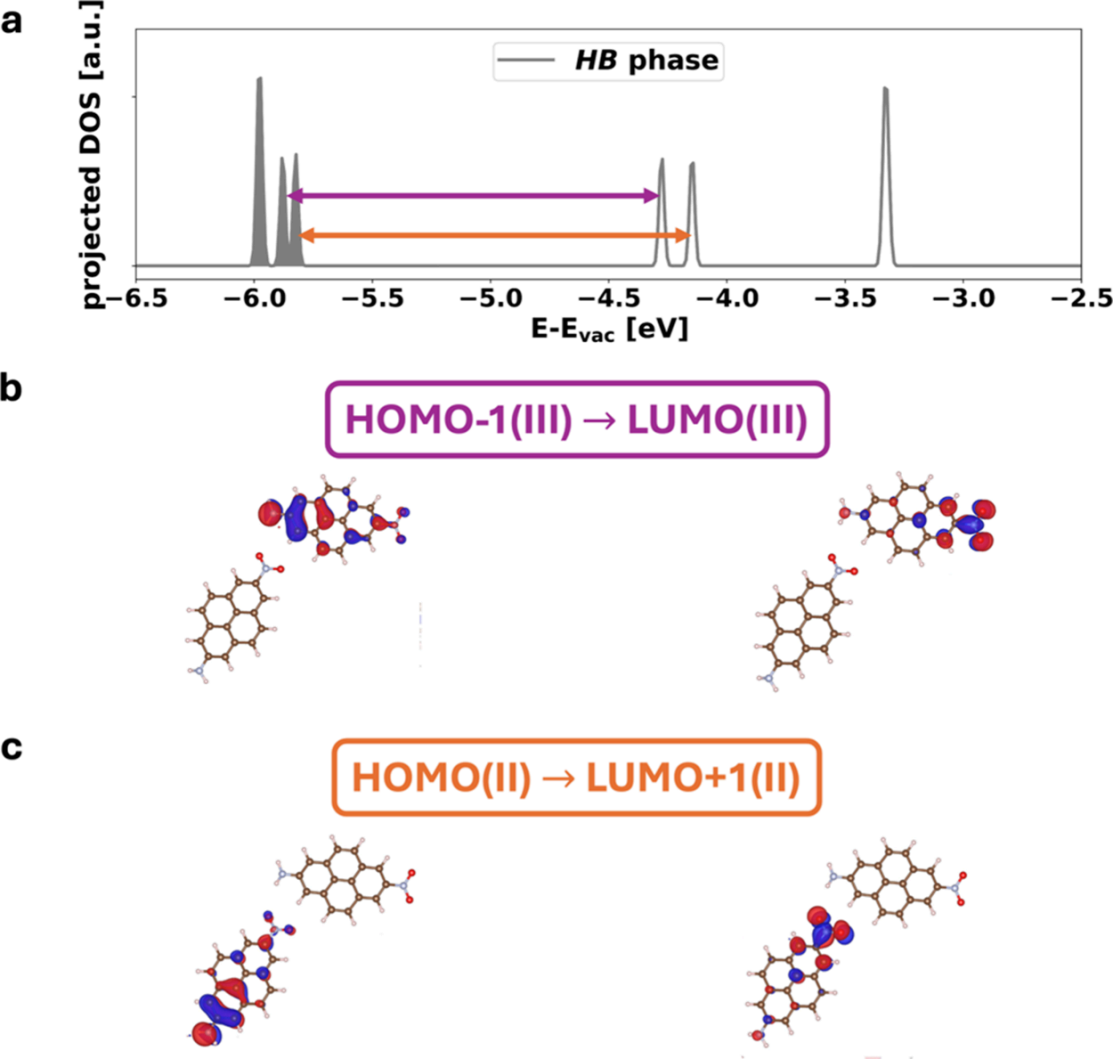
Panel a shows the density of states (DOS) of the system projected onto the molecular film of the ***HB*** phase on NaCl. The colored arrows correspond to the first two optically active transitions. The occupied states are indicated by the filled areas. Intensities of the peaks can be compared only within one DOS. Purple indicates the first transition from the HOMO–1­(III) to the LUMO­(III) localized on adsorption geometry III (panel b). The orbitals, participating in the second HOMO­(II)→LUMO+1­(II) transition, are localized on II (panel c) and indicated with an orange arrow in the projected DOS.

To get a deeper insight into the role of molecule–molecule interactions for the spectra, we performed calculations where we took the molecules within one unit cell of the two polymorphs and increased the distance between the building blocks (on a large supercell of NaCl, as shown in Figure S3). In the case of the ***BW*** phase, where there are 4 molecules in the unit cell (with two identical adsorption geometries, I and II), the molecules are separated in pairs at their inversion center (as shown in Figure 9a, lower panel).

**Figure 9:**
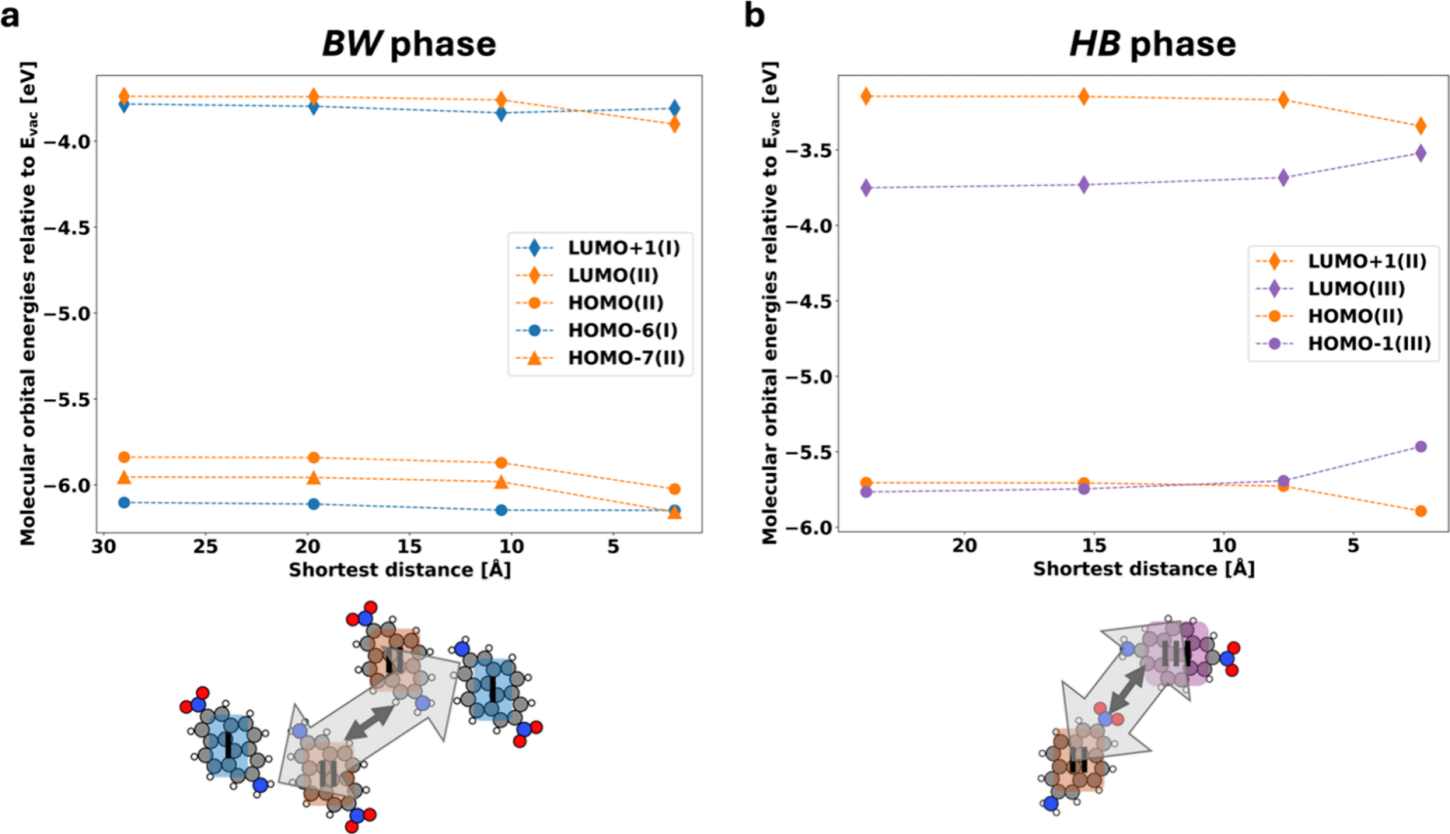
Energies of the molecular orbitals on NaCl localized on adsorption geometries corresponding to the first two optically active transitions (see Figures 7 and 8) over the shortest distance between the molecules forming the ***BW*** and ***HB*** phase, shown in panels a and b, respectively. The coloring of the lines is consistent with the adsorption geometries shown in the lower panel.

As Figure 9a shows, the orbital energies of the molecules on the “outside” of the dimers (which, here, were chosen to be the molecules on adsorption site I) are almost independent of the distance between the dimers. Conversely, for the “inner” molecules (adsorption site II), we see a notable downward shift of all relevant orbitals (LUMO, HOMO, and HOMO–7). Notably, the shift is almost the same for all of those orbitals. This indicates that the physical origin of this shift is of an electrostatic nature^43^ (i.e., the fact that the molecules feel the electrostatic potential arising from the nature of the adjacent molecule), rather than being related to the wave function overlap between the molecules. The electrostatic potential of one nitropyreneamine molecule can be found in the Supporting Information. The wave function overlap should be different for each orbital and, furthermore, result in a splitting into bonding and antibonding contributions (i.e., linear combinations between the orbitals of the two molecules with different relative phases), which we, however, do not observe.

This interpretation is further corroborated by Figure 9b, which shows the orbital energies as a function of the distance in the HB phase. Here, upon closing the distance between the molecules, we find a pronounced downward shift in energy for both the HOMO and LUMO for II and a notable upward shift for the HOMO and LUMO of III. In other words, for a given molecule, both the occupied and the unoccupied orbitals shift in the same direction, but the sign of the shift differs between the two adsorption sites. Interestingly, we also find that for adsorption site III, the magnitude of the shift differs between HOMO and LUMO, resulting in a reduction of the band gap of around 0.07 eV. We attribute the different sign of the shift of the orbitals to the fact that molecule III is situated close to the negatively charged NO_2_ group of the adjacent molecules (i.e., to the negative side of the dipole), while conversely, II is located close to the positive NH_2_ group of the adjacent molecule, i.e., the positive side of the dipole. The different magnitude of the shift between HOMO and LUMO of II then presumably originates from the different localization of HOMO and LUMO (see Figure 8b,c), experiencing different net electrostatic potentials.

We note that in Figure 9, we only analyze a subset of the interactions that occur within a monolayer, which has many more intermolecular interactions (see Figure S3 in the Supporting Information). However, even in the full monolayer, there appears to be hardly any energy shift due to wave function overlap. If there were, the band structure of the HOMO or LUMO-derived bands should show considerable dispersion. However, they are almost completely flat, showing a negligible bandwidth of 15 meV or less, as reported in Table S1 in the Supporting Information. Therefore, it must be the complex electrostatic landscape in a full monolayer that arises from the relative position and orientation relative to the dipoles of the various molecules that leads to the observed band gap reduction of around 0.2 eV for the ***BW*** phase and around 0.3 eV for the HB compared to the individual molecules on NaCl (Figure 10).

**Figure 10:**
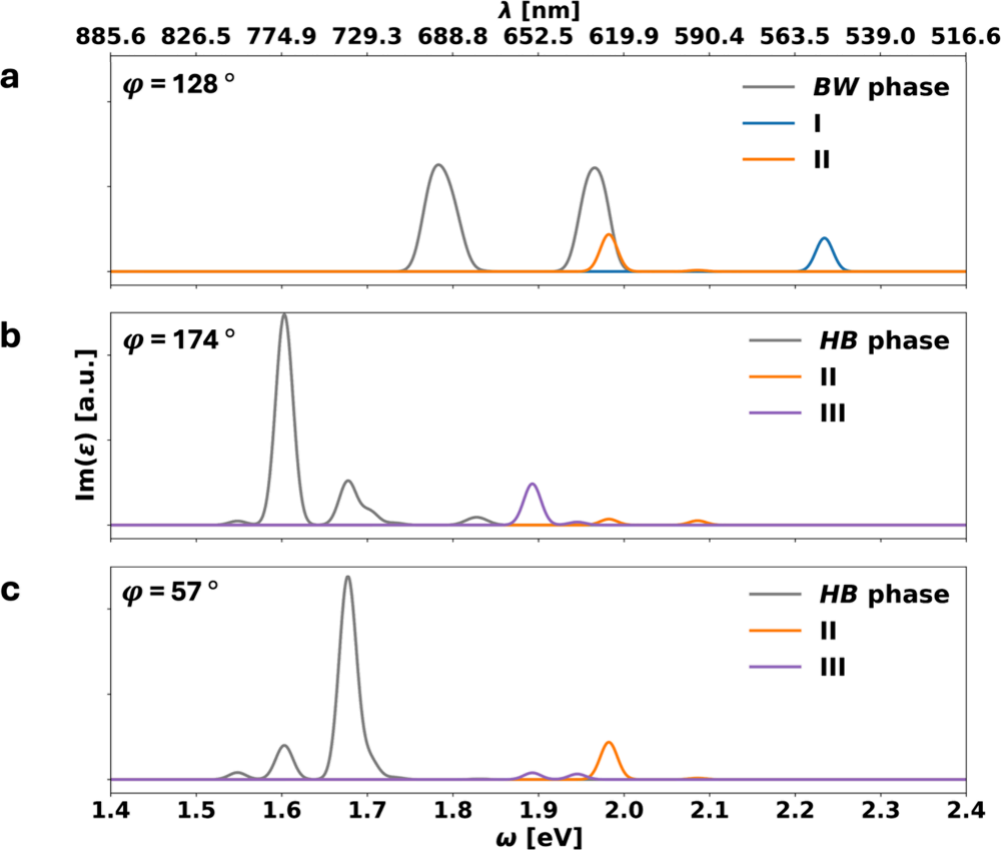
Imaginary part of the dielectric function Im­(ε) plotted for the ***BW*** (panel a) and ***HB*** phase (panels b and c) on NaCl(100) together with the isolated constituent adsorption geometries. For the ***HB*** phase, we are considering the two polarizations due to the large difference in orientation of the two adsorption geometries II and III, as in Figure 4. The polarization angle φ is defined as shown in Figure 3 in the illustration on the upper right.

## Conclusions

In this computational study, we explore the differences in the light absorption between two prototypical polymorph phases of a monolayer of the dipolar molecule 2-nitro-pyrene-7-amine on NaCl(100). Our calculations are based on the calculation of the dielectric function using density functional theory. Although this, technically, does not incorporate many-body responses such as the screening of the molecular exciton by the surface, we expect that these effects are very similar between the two polymorphs, and thus, the differences observed between the two polymorphs remain qualitatively unaffected.

For our system, we observe that upon adsorption of the molecule on NaCl, the first optically allowed transition splits into two different absorption bands, and that the excitation energies differ by around 0.2 eV between the two polymorphs. We demonstrate that this difference cannot simply be explained by Kasha’s theory or Davydov splitting. Rather, they occur as a result of a variety of interactions. While the impact of the geometry distortion upon adsorption on the surface is relatively small, the electronic interaction with the chemically inert NaCl surface leads to a substantial shift of the orbital energies, which depends very sensitively on the exact adsorption site and is a result of the localization of the different orbitals and their position relative to the positively/negatively charged ions of the surface. The intermolecular interaction in a closed monolayer of molecules leads to a further reduction of the band gap, which appears to be caused mostly by electrostatic interactions, i.e., the position of one molecule relative to the dipole moments of the other molecules. Conversely, notable effects from the coupling of transition dipole moments or from wave function overlap, which would have been evident from the formation of energetically well-separated bonding/antibonding linear combinations of the orbitals or, at least, a notable band dispersion in the monolayer, are almost completely absent. The magnitude of the different effects is summarized in Figure 11.

**Figure 11:**
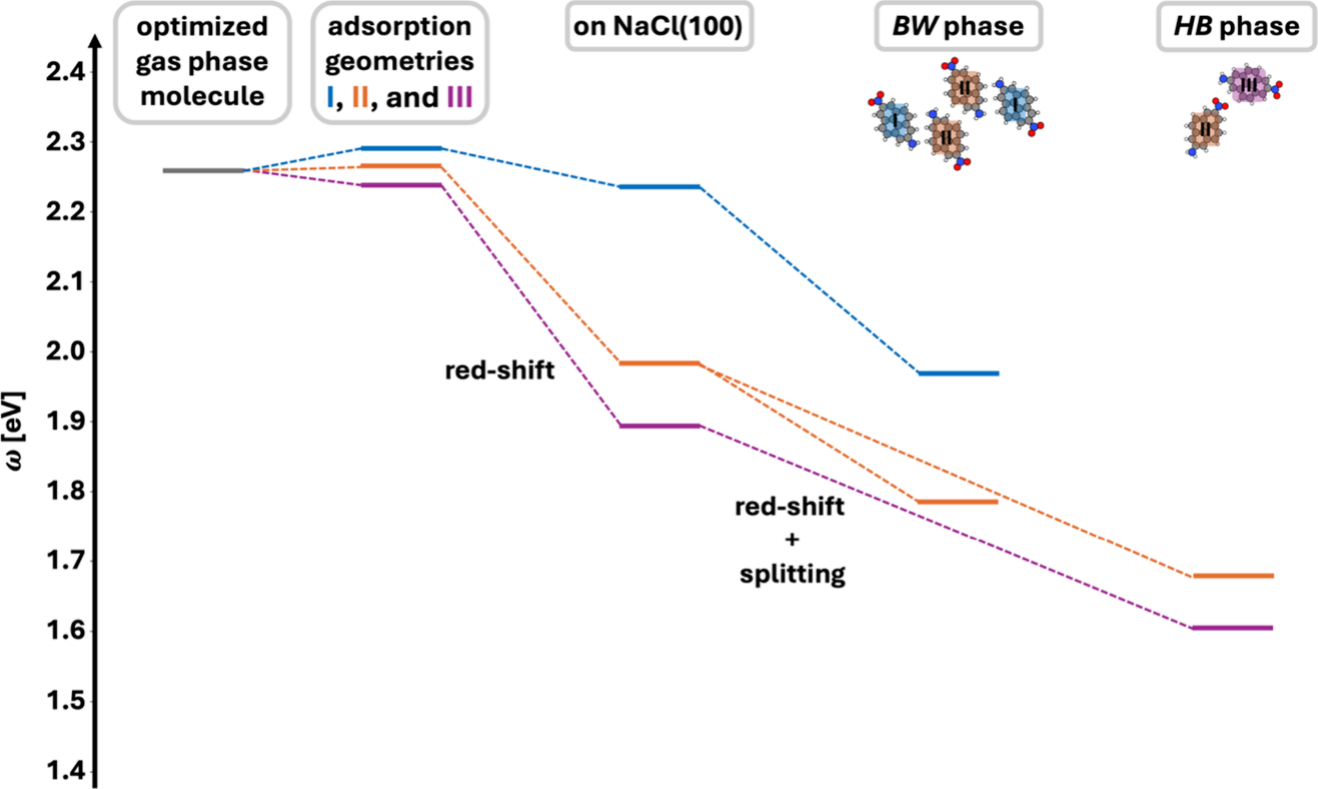
Summarizing plot of the first optically active transition energies for (i) the isolated adsorption geometries in the gas phase, (ii) the isolated adsorption geometries on NaCl(100), and (iii) the two polymorphs on NaCl, i.e., ***BW*** and ***HB*** phase. These demonstrate the effects of (i) the geometric distortion of each adsorption position, (ii) the molecule–substrate interactions, and (iii) molecule–molecule interactions, respectively.

## Computational Methods

The density functional theory calculations were performed using the FHI aims code^44^ with the “tight” basis set defaults as provided with the code and a Gaussian broadening of the states of 0.1 eV. In order to correctly describe the interaction between the organic molecule and an insulating layer, a GGA-PBE functional^45^ is used in combination with the nonlocal many-body dispersion (MBD-NL) van der Waals correction.^46,47^ The geometry optimizations were performed until the forces fell below 0.005 eV Å^–1^ for the isolated gas phase molecule. Likewise, to determine possible adsorption sites on NaCl, the molecule was placed on NaCl(100) in a (approximately 30 × 30 × 50) Å unit cell, corresponding to a 7 × 7 supercell of the substrate. Starting from 3 distinctly different adsorption positions (i.e., Na-top site, Cl-top site, and bridge site) and 6/12 different rotations for each top/bridge position, they were relaxed until the forces acting on each atom fell below 0.01 eV Å^–1^. For these calculations, a 4 × 4 × 1 Gamma-centered k-grid was employed. The 7 resulting inequivalent adsorption geometries (shown in the Supporting Information Figure S2) were subsequently used in the machine learning-based structure search algorithm SAMPLE^41^ (see below) to build the polymorph candidates for the structure search.

The SAMPLE code is an algorithm specifically developed for inorganic–organic interfaces. It uses a so-called “building-block approach” to create polymorph candidates. To do so, it generates differently sized supercells of the substrates and places as many molecules as possible in them, using the adsorption geometries of isolated molecules (as determined above) as building blocks. For this study, this generated a total of 5,573,279 polymorph candidates of nitropyreneamine on NaCl(100) by allowing NaCl supercells containing up to 29 NaCl unit cells, up to 4 molecules per supercell, and a minimum distance of 1.09 Å between the closest atoms of the molecules.

As the next step, SAMPLE utilizes a d-optimal drawing algorithm to determine a training set of structures. The energies of these structures are evaluated with dispersion-corrected DFT as described above. It then uses Bayesian linear regression to fit a simple energy model of the form

$$E_{\mathrm{config}}=\sum_{g}n_{g}U_{g}+\sum_{p}n_{p}V_{p}$$ 1

where *U*_*g*_ are one-body terms (physically corresponding to the molecule–substrate interaction) and *V_p_* are two-body terms (physically corresponding to the interaction between molecules). The values *n*_*g*_ and *n*_*p*_ define how often these interactions occur in a given structure. With a training set of 300 structures, which we provide via the NOMAD database under doi 10.17172/NOMAD/2025.07.15-1, we can predict the adsorption energies of all 5.5 million candidates with a leave-one-out cross-validation error of 11 meV. All employed hyperparameters are listed in the Supporting Information. A detailed explanation of the SAMPLE approach, which is available free of charge online at www.map-design.tugraz.at, is provided by Hörmann et al.^41^

For the present work, we only use two representative, low-energy polymorphs predicted by SAMPLE, which are the ***BW*** and the ***HB*** phase, as described in the main body of this manuscript. To analyze the optical properties of the ***BW*** and ***HB*** phases, single-point calculations were performed. To obtain accurate (transition) energies and resolve the reciprocal space accurately, a 4 × 8 × 1 and 5 × 13 × 1 k-grid is used, respectively. These correspond to a k-grid density between 0.05 and 0.06 Å^–1^. For the calculations of the dielectric function, the independent particle random phase approximation (IP-RPA) is used (employing the compute_dielectric flag in FHI aims) to output the whole dielectric tensor components, setting ω_max_ (the maximum transition energy) to 10 eV and *n*_ω_ to 40,000 (the number of points on which the dielectric function is evaluated), while choosing a Gaussian broadening of 0.01 eV.

We note in passing that the employed methodology (i.e., dispersion-corrected PBE calculations) is well-known to yield excellent agreement with experiment, especially regarding adsorption geometries and heights,^46^ dipole moments for molecules on substrates,^48,49^ or lattice constants (our determined lattice constant of NaCl, 5.62 Å, agrees within 0.4% with experimental values). Although PBE-derived band gaps are often smaller than experimentally determined optical excitations,^50,51^ relative shifts due to molecule–substrate and molecule–molecule interactions are generally very well reproduced.^42^

## Supplementary Material



## Notes

Band widths of ***HB*** and ***BW*** phase; Impact of polarization direction of the incoming light; Visualization of adsorption geometries of isolated nitropyreneamine molecules on NaCl; Visualization of the geometries used for the distance dependence shown in Figure 9; Hyperparameters employed for SAMPLE (PDF)
