# Supplementary material for: Relating Structural and Optical Properties of Organic Thin Films on Chemically Inert Substrates
Source: ACS Omega. 2025 Sep 26;10(39):45103–12. doi: 10.1021/acsomega.5c03920 (PMC12508980; doi:10.1021/acsomega.5c03920)
Supplement: Supplementary file 1 [file ao5c03920_si_001.pdf]

Supporting Information for Relating structural and optical properties of organic thin films on chemically inert substrates

Nina Kainbacher[1,2], Peter Puschnig[1], and Oliver T. Hofmann[2]

1 Institute of Physics, University of Graz, NAWI Graz, Universitätsplatz 5, 8010 Graz, Austria

2 Institute of Solid State Physics, Graz University of Technology, NAWI Graz, Petersgasse 16/II, 8010 Graz, Austria

The band widths of the lowest unoccupied and highest occupied state are listed in Table S1 to support the localization of the states of the two polymorph phases.

Table S1: Band widths of the lowest unoccupied and highest occupied state for the two polymorph phases.

	BW phase	***HB*** phase
Lowest unoccupied state [eV]	0.010	<0.005
Highest occupied state [eV]	0.015	0.005

To get an understanding what influences the polarization angle has on the absorption spectra (i.e., imaginary part of the dielectric function $\Im(\varepsilon)$), we analyzed 2-nitro-pyrene-7-amine in gas phase. As depicted in Figure S1a, the polarization angle is taken in the xy-plane relative to the x-axis. The variation of the angle is reflected in a distinctive modulation of the intensity of the absorption peaks in the absorption spectrum. For illustration, we plot the intensity of the first optically active transition as a function of the polarization angle in a polar plot (Figure S1b). In panel c, we also show the unpolarized absorption spectrum of the molecule as a reference for the peak position of the first optically active transition at around 2.3 eV (654 nm).

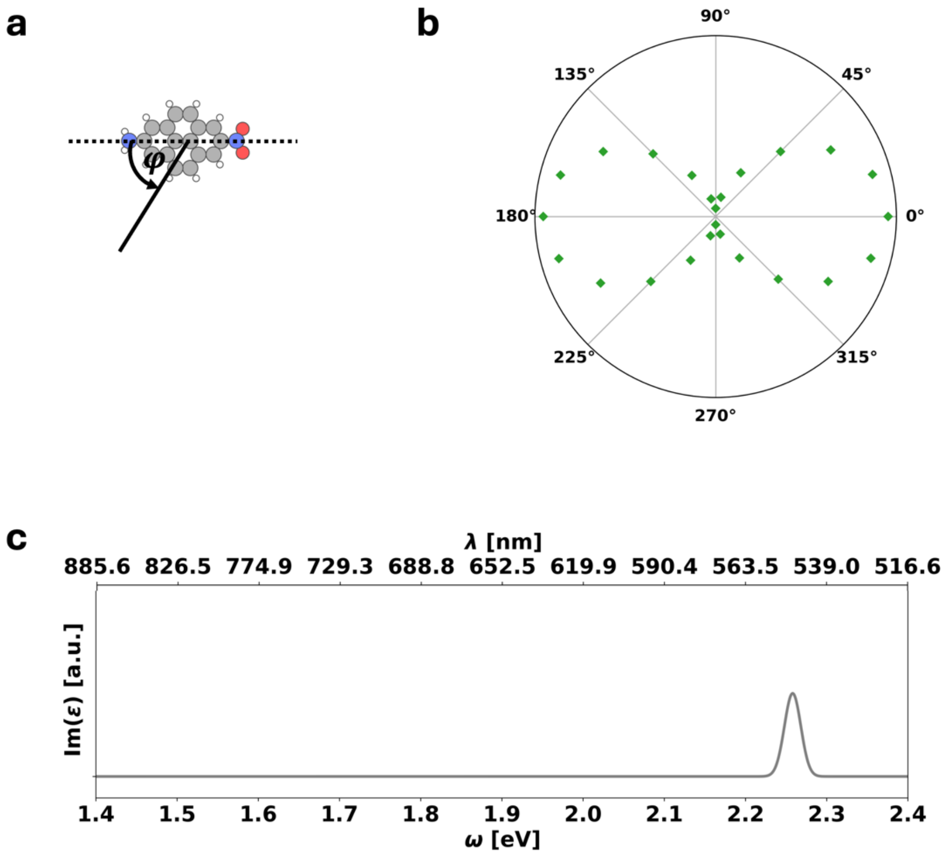

Figure S1: Absorption behavior of the gas phase molecule, i.e., monomer, and relationship between the polarization angle and the intensity of the first optically active transition. Panel a illustrates how polarization angle φ in the xy-plane is defined and panel b shows the polar plot of the intensities in 15 ° steps. In panel c, the unpolarized absorption spectrum of the single molecule with the first optically active transition is shown.

Here, the functional relationship between the polarization angle and the intensity clearly follows a $cos^2\varphi$ dependence. In the direction of the molecule's long axis the intensity is the largest, whereas, perpendicular to that (i.e., along its short axis) it is the smallest. Therefore, it is beneficial to only analyze and compare the absorption spectra polarized in the direction of the molecule's long axis. For this reason, we will consider the different polarization directions of the individual adsorption geometries in the polymorph's unit cell, as listed in Table S2.

*Table S2: Polarization angle along the molecule's long axis for each individual adsorption geometry of the two polymorph phases, the **BW** (I and II) and **HB** phase (II and III).*

	BW phase		***HB*** phase	
Adsorption geometry	**I**	**II**	**II**	**III**
Polarization angle φ [°]	128	128	174	57

Figure S2 shows all obtained unique adsorption geometries on NaCl(100) and their relative energies with respect to the adsorption geometry with the lowest (i.e., largest absolute) adsorption energy, which happens to be adsorption geometry **II**.

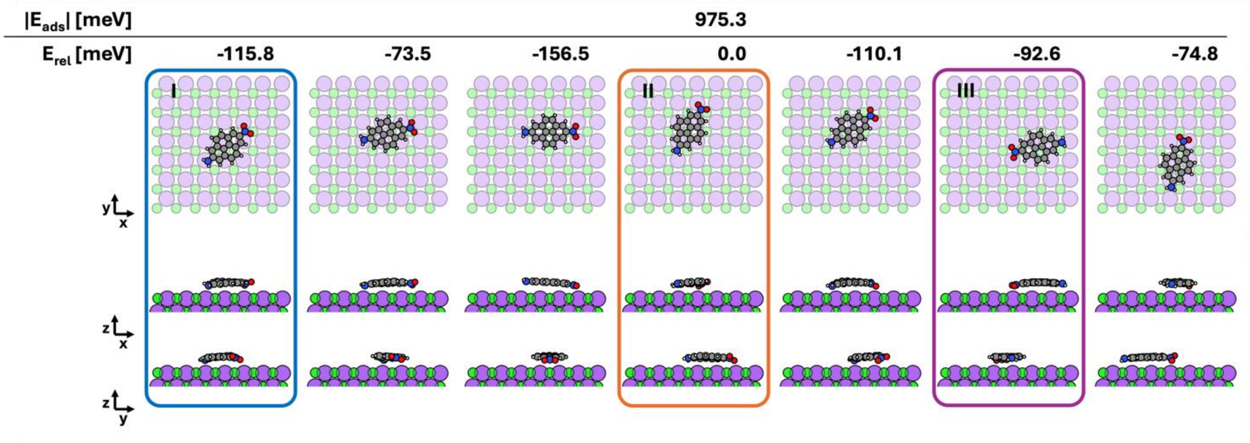

Figure S2: All unique adsorption geometries on NaCl(100). The adsorption geometries present in the two polymorph phases, **I**, **II**, *and* **III**, *are marked with their respective color (blue, orange, and purple).* $|E_{ads}|$ *is the absolute adsorption energy defined by* $|E_{ads}| = |E_{sys} – (E_{mol} + E_{sub})|$, *subtracting the energy of the relaxed molecule and substrate from the energy of the total system. The relative energy* E_{rel} *is then the difference between* $|E_{ads}|$ *of adsorption geometry* **II** (i.e., largest adsorption energy) *and* $|E_{ads}|$ *of the respective adsorption geometry.*

To analyze the molecule-molecule interactions, the molecule's contained in the unit cell of each polymorph phase was put in a large unit cell on NaCl(100) and the distance between the molecules was increased systematically. The unit cells are shown in Figure S3 and listed with the shortest distance d_{min} between the molecules.

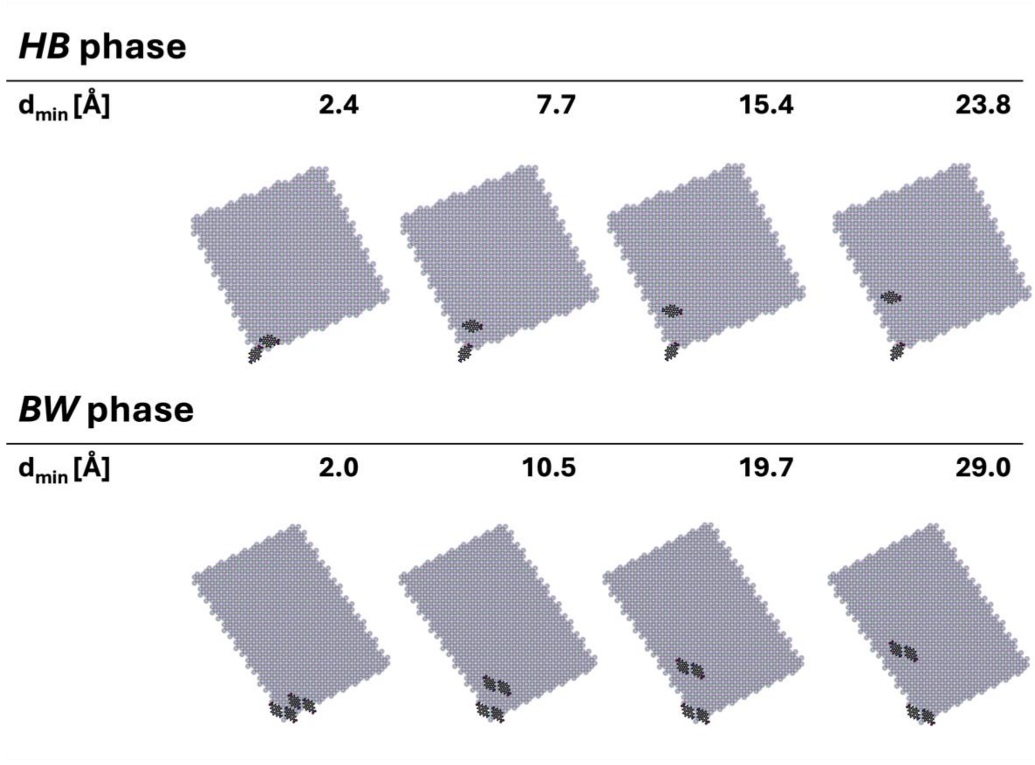

Figure S3: Visualization of each unit cell and the shortest distance d_{min} for the calculations of the orbital energies contained in the paper in Figure 9.

Figure S4 shows the electrostatic potential of the molecule over a range of 2 eV.

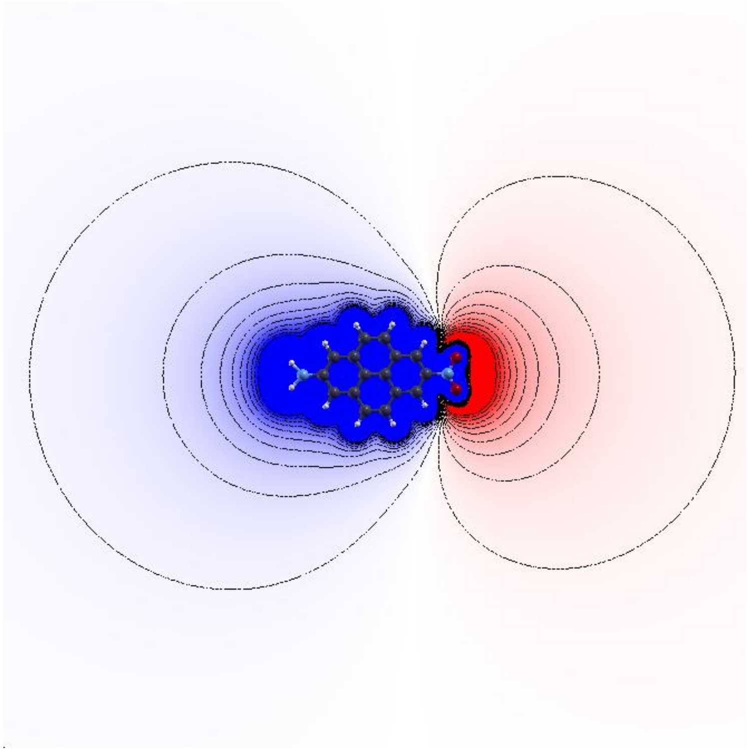

Figure S4: Electrostatic potential in plane of the nitropyreneamine molecule over a range of 2 eV. Each contour line corresponds to a shift of 0.1 eV.

Hyperparameters

The following hyperparameters have been employed within the SAMPLE code:

[Hyper Parameters]
E_ads_std = 0.2
E_pair_std = 0.2
DFT_noise = 0.01
decay_length = 5.0
decay_power = -2
correlation_length = 10.0[Feature Settings]
dmin = {('O', 'H'): 1.48, ('O', 'O'): 2.35, ('H', 'H'): 1.09, ('O', 'N'): 1.96, ('N', 'H'): 1.69, ('N', 'N'): 2.49, ('C', 'H'): 1.88, ('C', 'N'): 2.81, ('C', 'C'): 1.09, ('C', 'O'): 1.09}
